# Pressure-induced coherent sliding-layer transition in the excitonic insulator Ta_2_NiSe_5_

**DOI:** 10.1107/S2052252517018334

**Published:** 2018-01-26

**Authors:** Akitoshi Nakano, Kento Sugawara, Shinya Tamura, Naoyuki Katayama, Kazuyuki Matsubayashi, Taku Okada, Yoshiya Uwatoko, Kouji Munakata, Akiko Nakao, Hajime Sagayama, Reiji Kumai, Kunihisa Sugimoto, Naoyuki Maejima, Akihiko Machida, Tetsu Watanuki, Hiroshi Sawa

**Affiliations:** a Department of Applied Physics, Nagoya University, Nagoya, 464-8603, Japan; b University of Electro-Communications, Chofu, Tokyo 182-8585, Japan; c Institute for Solid State Physics (ISSP), University of Tokyo, 5-1-5 Kashiwanoha, Kashiwa, Chiba 277-8581, Japan; d Comprehensive Research Organization for Science and Society (CROSS), Tokai, Ibaraki 319-1106, Japan; e Photon Factory, IMSS, KEK, 1-1 Oho, Tsukuba, Ibaraki 305-0801, Japan; f Japan Synchrotron Radiation Research Institute (JASRI), SPring-8, Hyogo 679-5198, Japan; g Synchrotron Radiation Research Center (SRRC), National Institutes for Quantum and Radiological Science and Technology (QST), 1-1-1 Kouto, Sayo, Hyogo 679-5148, Japan

**Keywords:** inorganic materials, high-pressure single-crystal X-ray diffraction, excitonic insulators

## Abstract

The crystal structure of the excitonic insulator Ta_2_NiSe_5_ has been investigated under a range of pressures, as determined by the complementary analysis of both single-crystal and powder synchrotron X-ray diffraction measurements. The monoclinic ambient-pressure excitonic insulator phase II transforms upon warming or under a modest pressure to give the semiconducting *C*-centred orthorhombic phase I. At higher pressures (*i.e.* >3 GPa), transformation to the primitive orthorhombic semimetal phase III occurs. This transformation from phase I to phase III is a pressure-induced first-order phase transition, which takes place through coherent sliding between weakly coupled layers. This structural phase transition is significantly influenced by Coulombic interactions in the geometric arrangement between interlayer Se ions. Furthermore, upon cooling, phase III transforms into the monoclinic phase IV, which is analogous to the excitonic insulator phase II. Finally, the excitonic interactions appear to be retained despite the observed layer sliding transition.

## Abstract 2

Ta_2_NiSe_5_ has recently attracted interest as an excitonic insulator. In this paper, crystallographic parameters of three non-ambient phases are reported from across the *P*–*T* phase diagram. A reversible pressure-induced structural transition above 3 GPa is associated with the coherent sliding of weakly coupled layers.

## 1. Introduction   

In narrow-bandgap semiconductors and small band-overlap semimetals, bound electron–hole pairs spontaneously condense to drive the electronic state of the material towards a novel ground state, which is referred to as the excitonic insulator state (EI) (Mott, 1961 ▸; Halperin & Rice, 1968 ▸; Kohn, 1967 ▸; Jérome *et al.*, 1967 ▸; Bronold & Fehske, 2006 ▸). Although the idea of the EI was theoretically proposed in the 1960’s, few details regarding the physical properties of the EI phase have been determined due to the experimental identification of only a few materials that possess EI character (Bucher *et al.*, 1991 ▸; Wachter, 2001 ▸, 1995 ▸; Wilson, 1977 ▸; Cercellier *et al.*, 2007 ▸).

In recent years, however, the transition metal chalcogenide Ta_2_NiSe_5_ has attracted increasing attention in the context of EI behaviour. Previous reports have shown that Ta_2_NiSe_5_ crystallizes in a layered structure, where the stacks are bound through weak van der Waals interactions at ambient pressure and temperature (Sunshine & Ibers, 1985 ▸). In each of these layers, TaSe_6_ octahedral double chains parallel to the *a* axis interconnect with NiSe_4_ tetrahedral single chains along the *a* axis. Derived from this one-dimensional chain structure, the bottom of the conduction bands and the top of the valence bands consist mainly of Ta 5*d* and hybrid Se 4*p*–Ni 3*d* orbitals, respectively, which results in the formation of a one-dimensional electronic structure with a small band gap at the Γ point of the Brillouin zone (Canadell & Whangbo, 1987 ▸; Kaneko *et al.*, 2013 ▸). It has also been reported that Ta_2_NiSe_5_ exhibits a semiconductor-to-insulator transition at *T*_s_ = 328 K, which is accompanied by an orthorhombic-to-monoclinic second-order structural phase transition (Di Salvo *et al.*, 1986 ▸). However, the microscopic driving forces behind this semiconductor-to-insulator transition were not clear from the reported results, and so an additional study focusing on angle-resolved photoelectron spectroscopy was required to reveal anomalous flattening of the Ta_2_NiSe_5_ valence bands below *T*_s_, which confirmed the presence of the EI ground state (Wakisaka *et al.*, 2009 ▸). Recently, the presence of this EI phase within Ta_2_NiSe_5_ has been confirmed by employing alternative experimental methods, such as bandgap tuning by elemental substitution (Lu *et al.*, 2017 ▸) and exciton Fano resonance (Larkin *et al.*, 2017 ▸). It is noteworthy that Ta_2_NiS_5_ (Sunshine & Ibers, 1985 ▸), the isostructural compound of Ta_2_NiSe_5_, does not exhibit excitonic properties, thus Ta_2_NiSe_5_ is now seen as a potential platform for further study of the physical properties associated with EI behaviour.

**Figure 1:**
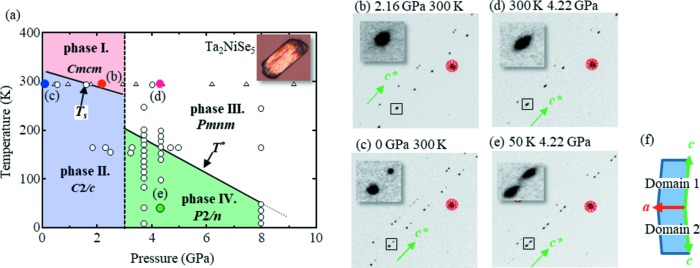
(*a*) Pressure–temperature phase diagram for Ta_2_NiSe_5_. A photographic image of the single crystal used for the high-pressure measurements is shown in the inset. Empty circles and triangles represent XRD measurement points using single-crystal and powder data, respectively. (*b*)–(*e*) Single-crystal XRD patterns at (*b*) 2.16 GPa, 300 K, (*c*) 0 GPa, 300 K, (*d*) 4.22 GPa, 300 K and (*e*) 4.22 GPa, 50 K. In each diffraction pattern, the peaks arranged along c* are derived from Ta_2_NiSe_5_, and the diffraction spots highlighted in red arise from single crystals of the diamond present in the DAC. (*f*) Schematic illustration of a monoclinic twin relationship between domains 1 and 2.

As the formation of excitons is strongly influenced by the carrier density, the electronic state can be investigated by controlling the electronic density state. One such method is the use of physical pressure to change the band-gap width. In this context, our preliminary high-pressure electrical resistivity measurements of Ta_2_NiSe_5_ produced a comprehensive pressure–temperature phase diagram that included the phase transitions and boundaries of the I–IV phases, in addition to a further high-pressure superconducting phase (Fig. 1 ▸ *a*; Matsubayshi *et al.*, 2018 ▸). This phase diagram also indicated that the phase transition to an EI state (phase II) at *T*_s_ was suppressed upon increasing the pressure. Furthermore, upon pressurization at room temperature (∼300 K), the semiconducting electrical resistivity of phase I undergoes phase transition to semimetal phase III at ∼3 GPa. Upon decreasing the temperature, an anomalous shoulder appears in the electrical resistivity at T* and a transition from phase III to phase IV is observed, while upon increasing the pressure, *T* ^*^ is suppressed to zero at ∼8 GPa and a superconducting phase emerges with a *T*_sc_ of ∼1.2 K. However, the microscopic origin of the phase transition at 3 GPa and the validity of the EI scenario even in the high-pressure phase remain unclear.

To better understand the physical properties of Ta_2_NiSe_5_ under pressure, investigation of its exact crystal structure is of particular importance. As interlayer bonding is generally significantly weaker than intralayer bonding in layered compounds, such as Ta_2_NiSe_5_, a large and anisotropic change in the crystal structure is expected upon the application of pressure.

Thus, we report herein our determination of the precise crystallographic parameters of Ta_2_NiSe_5_ in the phase I, III and IV regions *via* complementary analysis of both single-crystal and powder X-ray diffraction (XRD) patterns in high-pressure systems. In addition, we describe an anomalous structural phase transition which is associated with coherent sliding layers that originate from shifts in the interlayer Se ion arrangements. These results are expected to provide fundamental information regarding the electronic state of Ta_2_NiSe_5_ under high pressure.

## 2. Experimental   

The single-crystal of Ta_2_NiSe_5_ employed herein was synthesized *via* a chemical vapour transport method using I_2_ as the transport agent (Sunshine & Ibers, 1985 ▸). High-pressure single-crystal XRD measurements were performed on the BL22XU beamline at the SPring-8 facility (Watanuki *et al.*, 2007 ▸), Japan, using a wavelength λ = 0.4130 Å. Experiments were conducted using the same crystal while varying the temperature and pressure. The single crystal of Ta_2_NiSe_5_ (60 × 30 µm, 10 µm thickness, see inset of Fig. 1 ▸ *a*) was loaded into a hole (180 µm diameter) in an SUS gasket with helium as the pressure medium to give the *ac* plane perpendicular to the incident X-ray beam. The diamond anvil cell (DAC) was mounted in a closed-cycle helium refrigerator and the pressure was determined by the ruby luminescence method (Noack & Holzapfel, 1979 ▸; Nakano *et al.*, 2000 ▸). The sample pressure was regulated by a helium gas compression system. An imaging plate was used to obtain two-dimensional diffraction patterns *via* oscillation photography methods, and the resulting data were subsequently processed using software developed in-house (Sugawara *et al.*, 2018 ▸). Single-crystal XRD data were analysed using the *SHELXL97* least-squares program (Sheldrick, 2015 ▸). Single-crystal XRD experiments were also performed on the BL-8B beamline at the Photon Factory (PF), KEK (High-Energy Accelerator Research Organization), Japan, using a different single crystal from that employed on the BL22XU beamline at the SPring-8 facility. As crystallographic information regarding the orthorhombic phase (phase I) has not yet been reported, single-crystal XRD measurements were also carried out using the BL02B1 beamline at the SPring-8 facility (Sugimoto *et al.*, 2010 ▸) at ambient pressure and 400 K.

High-pressure powder XRD measurements were performed on the AR-NE1A beamline at the Photon Factory Advanced Ring (PF-AR), KEK, Japan, using a wavelength λ = 0.4179 Å. The powdered crystal was then prepared by grinding the single crystal, after which the powder was loaded into a hole (240 µm diameter) in an Re gasket. A 4:1 mixture of methanol–ethanol was used as the pressure medium and the pressure was determined using the ruby scale both before and after the diffraction measurements. Based on the results obtained for the single-crystal XRD analysis, Rietveld refinements (*GSAS* package; Larson & von Dreele, 1994 ▸) were carried out on the powder diffraction results under a range of pressures ≤9.17 GPa at room temperature. High-pressure and low-temperature powder XRD measurements were also performed on the BL02B1 beamline at the SPring-8 facility using a wavelength λ = 0.3101 Å. The powdered crystal was loaded into a hole (250 µm diameter) in an SUS gasket. A 4:1 mixture of methanol–ethanol was used as the pressure medium and an NaCl single crystal was also loaded as a pressure marker. The DAC was mounted in a closed-cycle helium refrigerator and the sample pressure was regulated by a helium gas compression system.

## 3. Results and discussion   

Following XRD measurements of the pressurized Ta_2_NiSe_5_ single-crystal sample at room temperature, three distinct phases (I, II and III) were identified and found to exhibit distinct crystal structures. Indexing of the XRD pattern recorded at 0 GPa and room temperature (Fig. 1 ▸ *c*, phase II) was carried out by assuming a single-crystal orientation, and thus monoclinic crystal symmetry was determined based on previous literature (Sunshine & Ibers, 1985 ▸). However, approximately half of the observed Bragg peaks could not be indexed, as they conflicted with the single-crystal approach, and so were attributed to the division of the crystal into several different domains as a result of structural phase transitioning (*T*_s_), as also observed in a previous report by Di Salvo *et al.* (1986 ▸). Considering these divided domains, it was then assumed that a different domain shared the *ab* plane along the single crystal (Fig. 1 ▸ *f*), and so a full index of the Bragg peaks could be completed. Note that the quasi-one-dimensional structure is preserved even in the presence of these domains. In this case, the crystal system and lattice parameters obtained showed a *C*-centred monoclinic system, with parameters *a* = 3.478 (1) Å, *b* = 12.800 (1) Å, *c* = 15.572 (1) Å and β = 90.52 (1)°.

Upon compression of the sample at 2.16 GPa, the number of Bragg peaks decreased by half, as illustrated in Fig. 1 ▸(*b*). This indicates that the two domains in phase II exhibiting monoclinic lattice symmetry have transitioned into a single domain with orthorhombic lattice symmetry, which is consistent with the phase diagram predicted by the electrical resistivity measurements. It therefore appeared that the *T*_s_ shifted below room temperature at an applied pressure of 2.16 GPa. The observed XRD pattern was then indexed relative to a *C*-centred orthorhombic system to give the following crystal structure parameters: *a* = 3.437 (1) Å, *b* = 12.559 (1) Å and *c* = 15.443 (1) Å. Upon examination of the crystal structure at this pressure, it was apparent that the structure was identical to that obtained at ambient pressure and high temperature (400 K, *T* > *T*_s_) by single-crystal X-ray diffraction measurements. The crystallographic data are shown in Table 1 ▸.

In contrast, the observed phase III had a distinctive crystal structure when compared with phases I and II. Following indexing of the XRD pattern of phase III at 4.27 GPa, a primitive orthorhombic lattice was obtained with parameters *a* = 3.437 (1) Å, *b* = 5.861 (1) Å and *c* = 15.512 (1) Å. It should be noted that the primitive lattice was observed only in the phase III measurements and contrasts with the *C*-centred lattice observed in phases I and II. More specifically, the length of the *b* axis is less than half that observed for phases I and II. In addition, the crystal structure in phase III has an *n*-glide plane perpendicular to the *b* axis since the generation rule (*h*0*l*: *h* + *l* = 2*n*) exists. Furthermore, following decompression to ≤2 GPa at room temperature, the crystal system and lattice parameters were restored to the values observed for phase II, thereby indicating that the pressure-induced structural phase transition is reversible.

**Figure 2:**
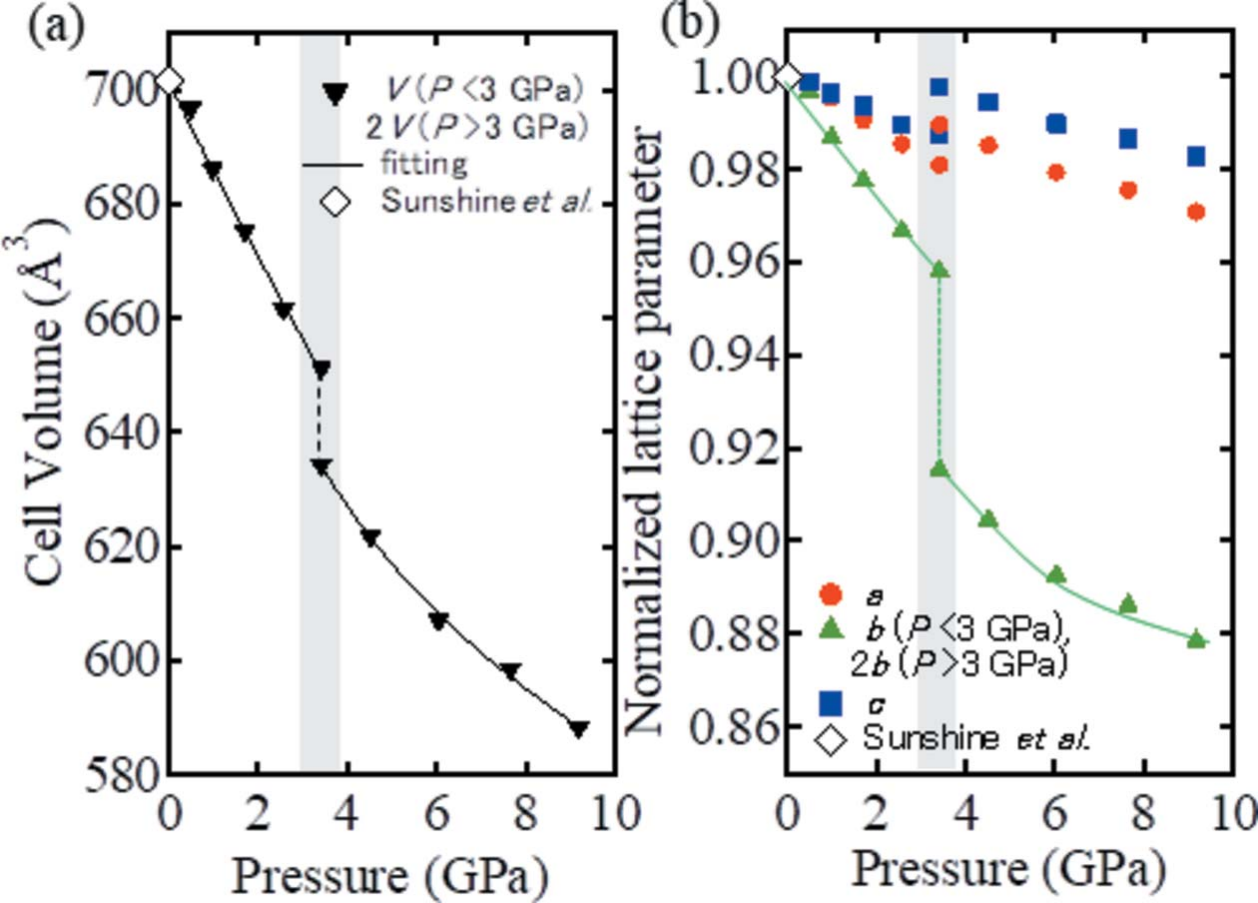
Pressure dependence of (*a*) the cell volume at room temperature and (*b*) the lattice parameters, normalized using the corresponding ambient lattice parameters (Sunshine & Ibers, 1985 ▸). Note that the *b* axis and cell volume are multiplied by 2 in the high-pressure phase region. Low- and high-pressure structures coexist within the shaded area at ∼3 GPa. The solid lines shown in panels (*a*) and (*b*) represent data fitting with respect to the equation of state and a guide for the eye, respectively.

Fig. 2 ▸ shows the pressure dependency of (*a*) the cell volume and (*b*) the normalized lattice parameters obtained at room temperature *via* Rietveld refinement of the powder XRD data. In this case, for comparison with phases I and II, both the *b* axis length and the cell volume for phase III were represented as double the observed values. The pressure dependence of this reduction in cell volume exhibited a discontinuity at 3 GPa. As phases II and III coexist in this portion of the plot (see Fig. 2 ▸), it is apparent that a first-order pressure-induced structural phase transition was observed at room temperature and 3 GPa. Furthermore, the proposed phase boundary produced a small gradient (d*P*/d*T*) relative to the first-order transition, while the cell volume decreased discontinuously. Thus, considering the Clausius–Clapeyron equation, d*P*/d*T* = Δ*S*/Δ*V*, the change in entropy within the system appears small in the context of the first-order transition. Moreover, in phases I and II, the lengths of the *a* and *c* axes were gradually reduced by the applied compression, whereas the length of the *b* axis decreased rapidly. Thus, comparison of phase III with phase I at 3 GPa indicates that the lengths of the *a* and *c* axes in phase III are only slightly longer (∼1%) than those of phase I. In contrast, the length of the *b* axis decreased by 6% during the transition from phase I to phase III, as will be discussed later in further detail.

The solid lines shown in Fig. 2 ▸(*a*) were then plotted following data fitting with a third-order Birch–Murnaghan equation of state (Birch, 1947 ▸): 
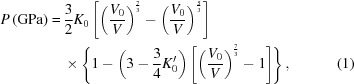
where *V*_0_ and *V* represent the unit-cell volume at ambient pressure and under applied pressure, respectively, and *K*_0_ and 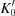 refer to the bulk modulus and its pressure derivative, respectively. Note that a pressure of 3.42 GPa was regarded as an ambient pressure point for fitting of the high-pressure region. From these calculations, the bulk modulus values (*K*_0_ and its pressure derivative 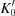) were determined to be 44 ± 3 and 48 ±5 GPa (K_0_), and 3.2 ± 0.6 and 6.7 ± 1.2 GPa (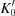), respectively, at room temperature both before and after the first-order structural transition. The value of 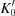, which was twice as large for phase III as for phase I, reflected the hardening effect of the bonding feature for the new crystal structure generated in phase III.

Table 1 ▸ shows the results of single-crystal X-ray diffraction analysis performed at 0, 2.16 and 4.22 GPa. It should be noted that, due to the limited aperture angle of the DAC, only 20% of the unique reflections were available for this analysis. Powder XRD was then carried out at ≤9.17 GPa at room temperature to obtain any unobserved reflections from the single-crystal XRD data. Upon conducting these measurements, a strong preferred orientation was observed due to the layered compound being placed in a uniaxial pressurized state within the DAC. To resolve this, Rietveld analysis served as a corrective technique to overcome the preferred orientation effect *via* spherical harmonic function calculations. The crystallographic parameters obtained from these powder analyses were consistent with the results of the previously shown single-crystal sample (Table 1 ▸). Further details and the corresponding powder XRD results are shown in Fig. S1 and Table S1 in the supporting information. The result of the band calculations will be reported elsewhere (Matsubayashi *et al.*, 2018 ▸).

**Figure 3:**
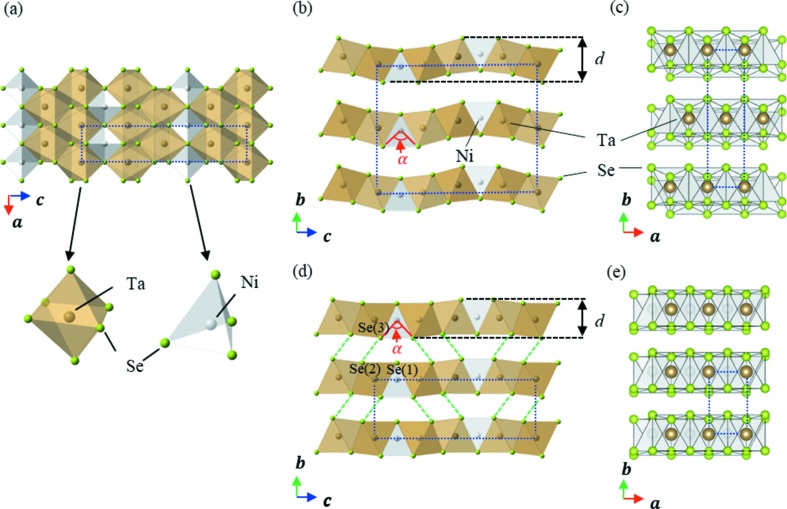
(*a*) The in-plane crystal structure viewed from the *b* axis (common to phases below and above 3 GPa). (*b*) and (*c*) The crystal structure of the phases below 3 GPa viewed from (*b*) the *a* axis and (*c*) the *c* axis. (*d*) and (*e*) The crystal structure obtained above 3 GPa viewed from (*d*) the *a* axis and (*e*) the *c* axis. The dotted blue lines indicate the unit cell for each structure at the given pressure, while α and *d* indicate the Se—Ni—Se bond angle and the layer thickness, respectively. The dashed green lines represent the Se(2)–Se(3) distance (see also Fig. 5 ▸ *a*).

Fig. 3 ▸ shows a comparison of the crystal structures of the phases observed at pressures less than and greater than 3 GPa, where it is apparent that the TaSe_6_ octahedral double chain and the NiSe_4_ tetrahedral single chains exist in a layered conformation in both cases (see Fig. 3 ▸ *a*). The most striking difference between these two crystal structures can be seen in their stacking patterns. More specifically, below 3 GPa two layers are present within the unit cell as a result of the *a*/2 lateral shift between neighbouring Ta_2_NiSe_5_ layers along their *b* axes to form the *C*-centred lattice (Fig. 3 ▸ *c*). However, above 3 GPa only a single layer is present within the unit cell, since the lateral shifting is eliminated to form the primitive lattice structure of the Ta_2_NiSe_5_ layers (Fig. 3 ▸ *e*). This suggests that layer sliding occurs as a result of the structural phase transition.

A possible concern arises in that the Ta_2_NiSe_5_ crystals could be broken or irreparably deformed during these drastic structural phase transitions. However, our results indicate that the crystal retained a nearly perfect periodicity throughout, as confirmed by the sharp diffraction spots shown in Fig. 1 ▸(*e*) and through a lack of observable diffusive scattering. We therefore propose that this layer sliding transition takes place both coherently and without any excessive generation of disorder in the crystal structure.

**Figure 4:**
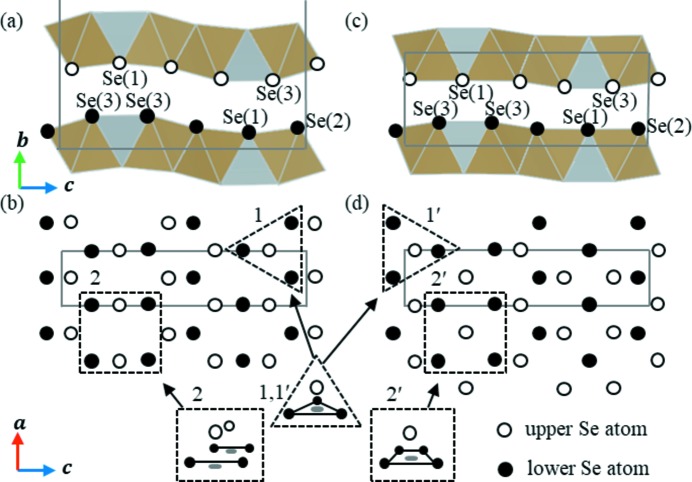
(*a*) and (*b*) Schematic representations of the arrangement of interlayer selenium atoms <3 GPa, viewed from the *a* and *b* axes, respectively. (*c*) and (*d*) As for panels (*a*) and (*b*) but at pressures >3 GPa. In panels (*a*) and (*c*) Se(1)–Se(3) represent three different crystallographic sites. Empty and filled circles indicate the upper- and lower-layer Se ions, respectively. Solid lines represent the size and shape of the unit cell. Sites 1 and 2 represent stable and unstable phase II sites, respectively, while sites 1′ and 2′ represent stable phase III sites under high pressure following phase transition.

Fig. 4 ▸ shows a schematic representation of the arrangement of Se ions present in Ta_2_NiSe_5_, where it is indicated that the upper and lower Se ions in the two stacking layers face one another at pressures less than and greater than 3 GPa. As shown in site 1 of Fig. 4 ▸(*b*), the lower three Se ions adopt a triangular arrangement, with an upper Se ion present at the centre of gravity. This site is regarded as a ‘hollow’ site and is considered a stable arrangement. In the case of site 2, as viewed from the *b* axis direction, an upper Se ion is located between two lower Se ions. From a three-dimensional viewpoint, the Se ion in the upper layer is located at a saddle point and is unstable under applied pressure.

Similarly, at site 1′ in phase III (see Fig. 4 ▸ *d*) a stable arrangement comparable with that of site 1 is observed. In this case, it is apparent that at site 2′ the four lower Se ions are in centroid positions and adopt a rectangular arrangement. This produces a stable conformation as a result of the ongoing pressure-induced structural phase transition, and so all Se ions are in a stable arrangement when Ta_2_NiSe_5_ adopts the phase III structure.

The change in this arrangement can be accounted for by the movement of all upper-layer Se ions along the *a* axis by a distance of *a*/2 relative to the lower layer Se ions. This is achieved by a lateral shift between neighbouring Ta_2_NiSe_5_ layers along the *a* axis by *a*/2, and is a direct representation of the layer sliding observed for Ta_2_NiSe_5_, which permits the transition between a *C*-centred lattice and a primitive lattice.

Based on these results, the isostructural compounds of Ta_2_NiSe_5_ may also exhibit such a layer sliding transition. For example, in the structure of Ta_2_NiS_5_, the Se ions of Ta_2_NiSe_5_ are fully replaced with S ions (Sunshine & Ibers, 1985 ▸) and so are also expected to exhibit a layer sliding transition. However, since the ionic radii of Se^2−^ and S^2−^ differ from one another, the critical pressure for each species is also likely to be different.

**Figure 5:**
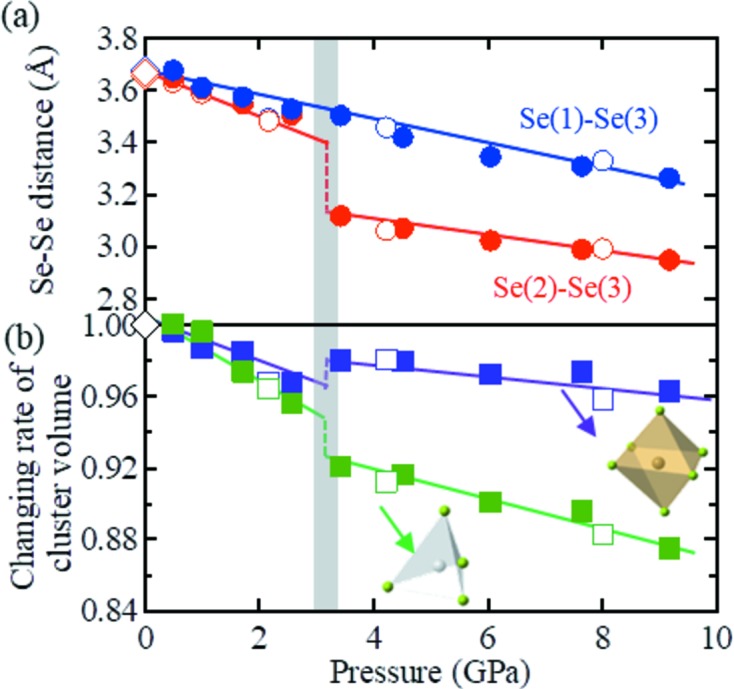
(*a*) Pressure dependence of the interlayer Se(1)–Se(3) (blue) and Se(2)–Se(3) (red) distances as determined by both single-crystal (empty circles) and powder (filled circles) XRD measurements. Se(1)–Se(3) represent three different crystallographic sites (as represented in Figs. 3 ▸ *d*, 4 ▸ *a* and 4 ▸ *c*). (*b*) Pressure dependence of the cluster volume of a Ta–Se octahedron (violet) and an Ni–Se tetrahedron (green). In each case, the empty and filled symbols represent the results of the single-crystal and powder XRD measurements, respectively. The blue, red, violet and green solid lines are guides for the eye. The experimental data points from a previous report at 0 GPa (Sunshine & Ibers, 1985 ▸) are plotted for comparison (diamonds).

Although the origin of the structural phase transition is identified as coherent layer sliding, the change in the crystal structure seems to be more complex. As shown in Fig. 5 ▸(*a*), the pressure dependence of the Se–Se distance between the layers varies greatly between Se(1)–Se(3) and Se(2)–Se(3) (see Fig. 3 ▸ *d*). More specifically, the Se(1)–Se(3) distance varies gradually at the transition pressure. However, the Se(2)–Se(3) distance exhibits a discontinuous jump at the transition pressure, and the short distance between Se(2) and Se(3) is represented by the green dashed line in Fig. 3 ▸(*d*). As the Se^2−^ species has an ionic radius of ∼2.0 Å, the short Se(2)–Se(3) distance of ∼3.0 Å indicates the presence of Se orbital hybridization between the layers. This is expected to increase significantly the dimensionality of the electronic states of Ta_2_NiSe_5_

In the case of a short Se(2)–Se(3) distance, a strong Coulombic repulsion is expected between Se(2) and Se(3). Thus, to reduce such repulsion, the bending of the layers, in which NiSe_4_ tetrahedral single chains are joined between two planar TaSe_6_ octahedral double chains, is significantly suppressed following the phase III transition, as shown in Fig. 3 ▸(*d*). This corresponds to a decrease in the layer thickness *d* (Figs. 3 ▸ *b* and 3 ▸ *d*) to ∼3.8 Å in phase III, which represents a decrease from 4.3 Å in phase I. This decrease in thickness can reach up to 12%, and can therefore shrink the *b* axis discontinuously at 3 GPa.

Furthermore, suppression of the layer thickness varies the bonding angle α of the Se(3)–Ni–Se(3) units (Figs. 3 ▸ *b* and 3 ▸ *d*), with a decrease to 85° being observed in phase III from a value of 96° in phase I. As a result, the Ni–Se tetrahedron is greatly distorted and its volume decreases discontinuously at 3 GPa (Fig. 5 ▸ *b*). Although this distortion increases the elastic energy of the lattice, it is assumed that the system obtains an energy gain by resolving the geometric instability of Se at the saddle point. In other words, the competition between the energy gain from resolving the geometric instability of the Se ions and the energy loss from distortions of the Ni–Se tetrahedra likely accounts for the reversibility of the pressure-induced structural phase transition.

It is therefore expected that the increased dimensionality of the crystal structure and the greatly distorted Ni–Se tetrahedron changes the electronic state of Ta_2_NiSe_5_ under high pressure, and so the temperature dependence of the electrical resistivity changes from semiconductor to semimetal character at 3 GPa. On the other hand, the Bragg peaks observed at 4.22 GPa and 50 K exhibit splitting that analogizes the phase transition to the excitonic insulator phase at *T*_s_ (Fig. 1 ▸ *e*).

**Figure 6:**
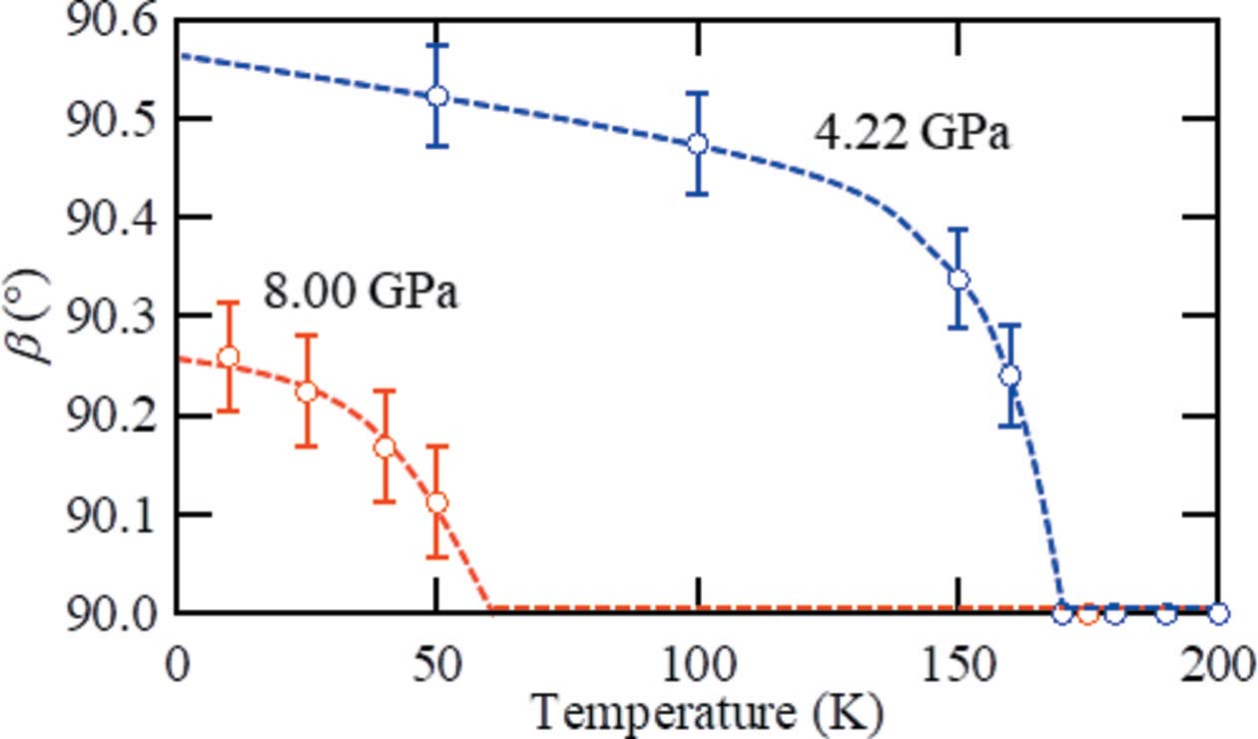
Temperature dependence of the monoclinic angle β at 4.22 and 8.00 GPa. The blue and red dashed lines are guides for the eye.

Assuming that two domains with monoclinic symmetry shared the *ab* plane, as in the case of phase II, a full index of the Bragg peaks could be completed and it was found that the monoclinic structure was present in phase IV. The temperature dependence of the monoclinic angle β is shown in Fig. 6 ▸, where it appears that the critical temperature of the structural phase transition corresponds to the T* value of the anomalous shoulder of the electrical resistivity. In addition, the saturation angle of β is 90.53 (6)°, which is comparable with that of a previous report at ambient pressure and temperature (Di Salvo *et al.*, 1986 ▸). Furthermore, as the extinction rule of the *n*-glide plane perpendicular to the *b* axis remained in phase IV, the space group was determined to be *P*2/*n*. The determined crystallographic parameters are shown in Table 1 ▸. As in phase III, the crystallographic parameters obtained by single-crystal XRD measurements were also examined by Rietveld analysis using powder XRD data measured on beamline BL02B1 at Spring-8. The anomaly at T* in the electrical resistivity tends to be smeared out with increasing pressure and is no longer detectable at 8 GPa. However, the structural transition takes place even at 8.0 GPa, below the temperature expected from an extrapolation of T* obtained from the electrical resistivity. The saturation angle of β was suppressed to 90.26 (6)°.

One plausible scenario to account for this structural phase transition at T* is that the excitonic phase transition exists even under high pressures. It is noteworthy that Se(1), whose 4*p* orbital forms a hybrid one-dimensional chain structure with the Ni 3*d* orbital (Kaneko *et al.*, 2012 ▸), is not significantly affected by the layer sliding transition when compared with Se(2) and Se(3). In addition, the pressure dependence of the Ta–Se octahedral cluster varies by only 4% even at 9 GPa, and the electronic state of Ta appears constant despite the compression. Therefore, the electronic structure composed of two Ta 5*d* one-dimensional chains and an Ni 3*d*–Se(1) 4*p* chain possibly remains within the structure, resulting in an excitonic phase transition even after the layer sliding transition has taken place.

Finally, we note that it is difficult to obtain an accurate band structure without the electron correlation effect in phases I and III (Matsubayashi *et al.*, 2018 ▸). Future studies are therefore expected to clarify this relationship between the electronic state of phase IV and the excitonic insulator state of phase II through accurate theoretical calculations using the crystallographic parameters and variations discussed herein.

## 4. Conclusions   

In summary, high-pressure X-ray diffraction measurements were carried out on the layered transition metal chalcogenide Ta_2_NiSe_5_ to characterize the crystallographic parameters for the four observed phases in the context of their semiconductor, excitonic insulator, semimetal and pseudogap properties. Using a prepared Ta_2_NiSe_5_ single crystal, both single-crystal and powder X-ray diffraction measurements were carried out under a range of pressures. Upon applying pressure at room temperature (∼300 K), three unique phases were observed, with transitions from *C*-centred monoclinic and orthorhombic structures to a primitive orthorhombic lattice system being recorded at high pressures. This reversible pressure-induced first-order structural phase transition was attributed to the varying spatial interactions of the Se ions at the different lattice sites of these phases, and was essentially attributed to coherent sliding between weakly coupled layers. After the layer sliding transition had taken place, an ortho­rhombic-to-monoclinic transition analogous to the ambient excitonic phase transition occurs at the corresponding temperature of the anomalous shoulder of the electric resistivity (*T* ^*^). It is implied that the excitonic phase transition also exists under high pressure. We therefore expect that our analysis and characterization of the phase transition phenomena of Ta_2_NiSe_5_ will lead to an ease of modelling, and will permit further studies of the potential properties of this material with regard to its pressure-dependent excitonic insulating and superconducting phases.

## Supplementary Material

Crystal structure: contains datablock(s) Ta2NiSe5_0GPa_400K, Ta2NiSe5_2p16GPa_300K, Ta2NiSe5_4p22GPa_200K, Ta2NiSe5_4p22GPa_50K. DOI: 10.1107/S2052252517018334/fc5022sup1.cif

Structure factors: contains datablock(s) Ta2NiSe5_0GPa_400K. DOI: 10.1107/S2052252517018334/fc5022Ta2NiSe5_0GPa_400Ksup2.hkl

Structure factors: contains datablock(s) shelxl. DOI: 10.1107/S2052252517018334/fc5022Ta2NiSe5_2p16GPa_300Ksup3.hkl

Structure factors: contains datablock(s) Ta2NiSe5_4p22GPa_200K. DOI: 10.1107/S2052252517018334/fc5022Ta2NiSe5_4p22GPa_200Ksup4.hkl

Structure factors: contains datablock(s) . DOI: 10.1107/S2052252517018334/fc5022Ta2NiSe5_4p22GPa_50Ksup5.hkl

Details of high-pressure single-crystal XRD measurement and Rietveld refinement. DOI: 10.1107/S2052252517018334/fc5022sup6.pdf

CCDC references: 1815855, 1815856, 1815857, 1815858

## Figures and Tables

**Table:** 

Pressure (GPa)	0	2.16	4.22	4.22
Temperature (K)	400	300	200	50
Phase	I	I	III	IV
Wavelength (Å)	0.2786	0.4130	0.4130	0.4130
Space group	*Cmcm*	*Cmcm*	*Pmnm*	*P*2/*n*
*a* (Å)	3.5029 (1)	3.437 (1)	3.437 (1)	3.437 (1)
*b* (Å)	12.8699 (5)	12.559 (1)	5.861 (1)	5.849 (11)
*c* (Å)	15.6768 (8)	15.443 (1)	15.512 (1)	15.512 (1)
β (°)	90	90	90	90.53 (1)
*V* (Å^3^)	706.74 (5)	656.96 (9)	312.5 (1)	311.8 (6)
*Z*	4	4	2	2
ρ_calc_ (g cm^−3^)	7.663	8.224	8.685	8.685
*F*(000)	1376	1376	688	688
(sinθ/λ)_max_ (Å^−1^)	1.25	0.77	0.77	0.77
*N*_Tot,obs_	32115	467	378	456
*N*_Uniq,obs_	3100	169	156	189
*N*_Parameters_	27	14	14	18
GOF	1.144	0.998	1.137	1.041
*R*_int_	3.73	3.10	4.75	6.94
*R*_1_, *R*_1_ [*F*_o_ > 4σ(*F*_o_)]	1.97, 2.38	4.33, 3.52	4.05, 3.45	4.75, 5.44
*wR*_2_	5.30	9.01	8.08	11.07
Δρ_max_, Δρ_min_	3.739, −2.617	2.033, −2.776	2.304, −2.305	1.91, −2.00

**Table:** 

	Wyckoff position	Position [*x*, *y*, *z*]	*B*_eq_ (Å^2^)
Ta_2_NiSe_5_ (400 K and 0 GPa)			
Ta	8*f*	[0.5, 0.221158 (6), 0.110222 (4)]	0.92 (1)
Ni	4*c*	[1, 0.20096 (3), 0.25]	1.05 (1)
Se(1)	8*f*	[0.5, 0.32679 (2), 0.25]	0.97 (1)
Se(2)	8*f*	[0, 0.354170 (15), 0.049338 (11)]	0.87 (1)
Se(3)	4*c*	[1, 0.080461 (15), 0.137726 (13)]	1.01 (1)
Ta_2_NiSe_5_ (300 K and 2.16 GPa)			
Ta	8*f*	[0.5, 0.22193 (28), 0.10988 (4)]	0.56 (2)
Ni	4*c*	[1, 0.20113 (127), 0.25]	0.61 (6)
Se(1)	8*f*	[0.5, 0.33106 (103), 0.25]	0.60 (5)
Se (2)	8*f*	[0, 0.35873 (76), 0.04875 (11)]	0.52 (3)
Se(3)	4*c*	[1, 0.07625 (79), 0.13832 (11)]	0.71 (4)
Ta_2_NiSe_5_ (200 K and 4.22 GPa)			
Ta	4*e*	[0.25, 0.4822 (6), 0.11304 (5)]	0.45 (2)
Ni	2*b*	[0.75, 0.471 (3), 0.25]	0.36 (6)
Se(1)	4*e*	[0.25, 0.2550 (17), 0.47 (3)]	0.47 (3)
Se(2)	4*e*	[0.75, 0.1770 (16), 0.14863 (12)]	0.52 (4)
Se(3)	2*a*	[0.75, 0.262 (2), 0.75]	0.51 (4)
Ta^2^NiSe^5^ (50 K and 4.22 GPa)			
Ta	4*g*	[0.7558 (5), 0.5182 (6), 0.8868 (1)]	0.27(4)
Ni	2*f*	[0.25, 0.5325 (28), 0.75]	0.22 (9)
Se(1)	4*g*	[0.2442 (15), 0.2566 (22), 0.9633(1)]	0.32 (5)
Se(2)	4*g*	[0.2465 (17), 0.8181 (21), 0.8515(1)]	0.36 (5)
Se(3)	2*f*	[0.75, 0.2594 (34), 0.75]	0.35 (6)
