# Supplementary material for: Pressure-induced coherent sliding-layer transition in the excitonic insulator Ta2NiSe5
Source: IUCrJ. 2018 Jan 26;5(Pt 2):158–65. doi: 10.1107/S2052252517018334 (PMC5947720; doi:10.1107/S2052252517018334)
Supplement: Supplementary file 6 [file m-05-00158-sup6.pdf]

IUCrJ

Volume 5 (2018)

Supporting information for article:

Pressure-induced coherent sliding transition in the excitonic insulator Ta2NiSe5

Akitoshi Nakano, Kento Sugawara, Shinya Tamura, Naoyuki Katayama, Kazuyuki Matsubayashi, Taku Okada, Yoshiya Uwatoko, Kouji Munakata, Akiko Nakao, Hajime Sagayama, Reiji Kumai, Kunihisa Sugimoto, Naoyuki Maejima, Akihiko Machida, Tetsu Watanuki and Hiroshi Sawa

S1. Details of Rietveld refinements

Rietveld analysis up to 9.17 GPa was succeeded with the isostructural model of single XRD result of 4.22 GPa, 200 K and corresponding result is presented in Table S1. However, the scattered intensity was highly affected by preferred orientation effect. Considering $(I_{010}/LP)/(I_{200}/LP)$, which is calculated from the observed intensities of powder X-ray diffraction data, is about 0.06 (I_{hkl} is the observed intensity, L is the Lorentz factor and P is the polarization factor), whereas the calculation of $|F_{010}|^2/|F_{200}|^2$ from the structural model is about 0.34 (F_{hkl} is the structure factor).Therefore, the structure factor ratio of (010) plane to (200) plane becomes less than one fifth in powder XRD experiments so as to need a correction of the intensity in Rietveld analysis. We carried out the correction by using *GSAS* package; the 6th order spherical harmonic function was applied with a texture index of 1.68. As a result, the observed and calculated intensity show a good agreement (Fig. S1), with agreement factors of R_{wp}=0.0350, R_p=0.0246 and R_{F2}=0.0375.

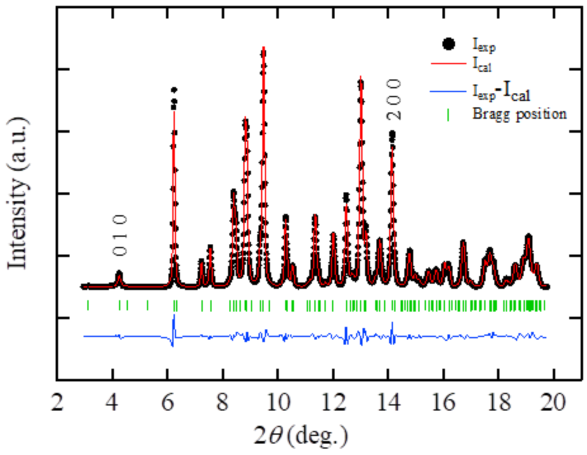

Figure S1 X-ray diffraction pattern of Ta_2NiSe_5 at 9.17 GPa. The interval of 2θ between measurement points is 0.01 degree.

Table S1 Results of the Rietveld analysis at various pressures up to 9.17 GPa

Pressure (GPa)	0.49	0.99	1.71	2.56	3.42	4.51	6.04	7.64	9.17
temperature (K)	300	300	300	300	300	300	300	300	300
wavelength (Å)	0.4178	0.4178	0.4178	0.4178	0.4178	0.4178	0.4178	0.4178	0.4178
phase	II	II	I	I	III	III	III	III	III
Space group	*C*2/*c*	*C*2/*c*	*Cmcm*	*Cmcm*	*Pmnm*	*Pmnm*	*Pmnm*	*Pmnm*	*Pmnm*
a (Å)	3.48790(5)	3.476893(8)	3.4635(1)	3.44509(8)	3.45970(10)	3.44447(9)	3.42395(8)	3.41083(9)	3.3943(1)
b (Å)	12.7875(95)	12.6616(11)	12.5422(18)	12.4023(14)	5.8720(6)	5.8024(6)	5.7257(5)	5.6839(6)	5.6362(5)
c (Å)	15.6210(2)	15.5841(3)	15.5401(4)	15.4805(3)	15.6070(4)	15.5541(4)	15.4808(3)	15.4336(1)	15.3721(3)
β (°)	90.383(2)	90.326(3)	90	90	90	90	90	90	90
V (Å^3)	696.707(48)	686.051(56)	675.055(9)	661.436(68)	317.065(3)	310.868(29)	303.497(25)	299.21(26)	294.08(25)
Z	4	4	4	4	2	2	2	2	2
F(000)	1376	1376	1376	1376	688	688	688	688	688
$(\sin\theta / \lambda)_{max}$ (Å^{-1})	0.81	0.81	0.81	0.81	0.81	0.81	0.81	0.81	0.81
Nobs	251	247	160	153	130	130	130	129	133
GOF	2.7	4.1	5.4	4.1	3.7	4.3	3.6	3.6	3.1
wR_p	0.0261	0.0445	0.0572	0.0454	0.0389	0.0486	0.0407	0.0405	0.0246
R_{F2}	0.0465	0.0397	0.0532	0.0396	0.0522	0.0443	0.0306	0.0636	0.0375

S2. Determination of the crystal lattice symmetry of phase III & IV

Synchrotron single crystal XRD experiment was carried out at the KEK PF BL8B using a wavelength λ=0.6888Å, with the temperature regulated by a cryostat. Diffracted intensities were collected on an IP detector. The single crystal of Ta_2NiSe_5 was loaded into a hole of 180 μm in diameter in a SUS gasket. The pressure was determined by the lattice constant of NaCl crystalline in a 4:1 methanol-ethanol mixture used as the pressure medium. The determined value of the applied pressure was 3.8 GPa.

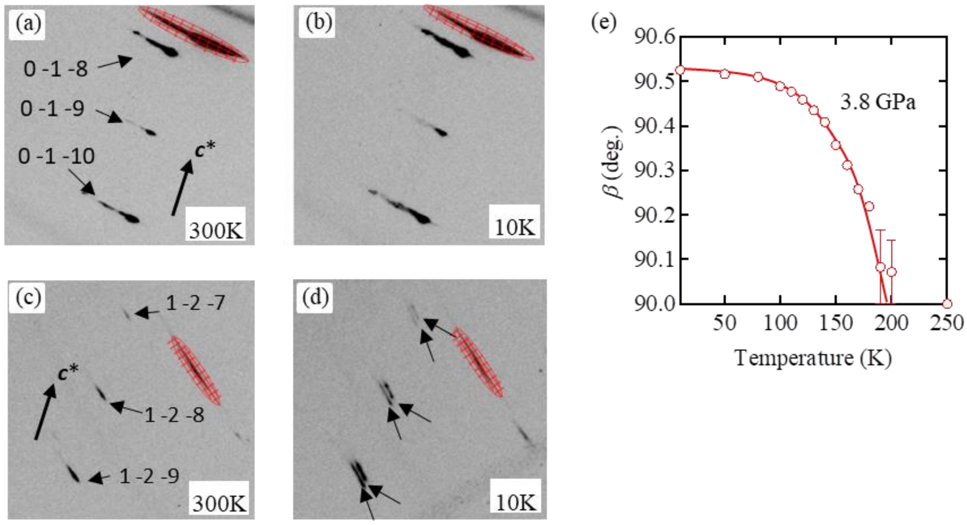

Figure S2 Single-crystal XRD patterns at 3.8 GPa for ((a) and (b)) 0 *k l* peaks and ((c) and (d)) *h k l* peaks. Red meshed areas indicate Bragg peaks from NaCl implemented as a pressure marker. (e) Temperature dependence of the monoclinic angle β at 3.8 GPa.

Figures S2(a) and (b) show XRD profiles of Miller indices 0 *k l* obtained at 300 K and 10 K, respectively. These Bragg peaks show no signs of splitting or broadening down to 10 K. In contrast, *h k l* peaks splits into double separate reflections upon decreasing temperature, as shown Figs. S2(c) and (d). This result indicates that the orthorhombic symmetry at phase III is lowered to monoclinic symmetry at phase IV. The transition temperature estimated from the temperature dependence of the monoclinic angle β (Fig.S2(e)) is about 200 K and it is likely to be consistent with the electrical resistivity measurement.
